# Interleukin-6 Mediates Epithelial–Stromal Interactions and Promotes Gastric Tumorigenesis

**DOI:** 10.1371/journal.pone.0060914

**Published:** 2013-04-12

**Authors:** Hiroto Kinoshita, Yoshihiro Hirata, Hayato Nakagawa, Kei Sakamoto, Yoku Hayakawa, Ryota Takahashi, Wachiko Nakata, Kosuke Sakitani, Takako Serizawa, Yohko Hikiba, Masao Akanuma, Wataru Shibata, Shin Maeda, Kazuhiko Koike

**Affiliations:** 1 Department of Gastroenterology, Graduate school of Medicine, The University of Tokyo, Tokyo, Japan; 2 Division of Gastroenterology, Institute for Adult Diseases, Asahi Life Foundation, Tokyo, Japan; 3 Department of Gastroenterology, Yokohama City University, Yokohama, Japan; Technische Universität München, Germany

## Abstract

Interleukin-6 (IL-6) is a pleiotropic cytokine that affects various functions, including tumor development. Although the importance of IL-6 in gastric cancer has been documented in experimental and clinical studies, the mechanism by which IL-6 promotes gastric cancer remains unclear. In this study, we investigated the role of IL-6 in the epithelial–stromal interaction in gastric tumorigenesis. Immunohistochemical analysis of human gastritis, gastric adenoma, and gastric cancer tissues revealed that IL-6 was frequently detected in the stroma. IL-6–positive cells in the stroma showed positive staining for the fibroblast marker α-smooth muscle actin, suggesting that stromal fibroblasts produce IL-6. We compared IL-6 knockout (IL-6^−/−^) mice with wild-type (WT) mice in a model of gastric tumorigenesis induced by the chemical carcinogen N-methyl-N-nitrosourea. The stromal fibroblasts expressed IL-6 in tumors from WT mice. Gastric tumorigenesis was attenuated in IL-6^−/−^ mice, compared with WT mice. Impaired tumor development in IL-6^−/−^ mice was correlated with the decreased activation of STAT3, a factor associated with gastric cancer cell proliferation. *In vitro,* when gastric cancer cell line was co-cultured with primary human gastric fibroblast, STAT3–related genes including COX-2 and iNOS were induced in gastric cancer cells and this response was attenuated with neutralizing anti-IL-6 receptor antibody. IL-6 production from fibroblasts was increased when fibroblasts were cultured in the presence of gastric cancer cell–conditioned media. IL-6 production from fibroblasts was suppressed by an interleukin-1 (IL-1) receptor antagonist and siRNA inhibition of IL-1α in the fibroblasts. IL-1α mRNA and protein were increased in fibroblast lysate, suggesting that cell-associated IL-1α in fibroblasts may be involved. Our results suggest the importance of IL-6 mediated stromal-epithelial cell interaction in gastric tumorigenesis.

## Introduction

Gastric cancer is a leading cause of cancer-related death [1]. The global incidence of gastric cancer was estimated to be 934,000 cases in 2002; 56% of new cases occurred in East Asia, 11% of which were in Japan [2]. Despite recent advances in combination chemotherapies [3], the outcome of unresectable gastric cancer remains poor, and new treatments, including molecularly targeted therapies, are urgently needed. Mutations and amplifications of certain kinases have been reported to be associated with human gastric carcinogenesis [4]. Nevertheless, trastuzumab, a monoclonal antibody that acts on the HER2/neu (erbB2) receptor, is currently the only molecularly targeted drug that is used against gastric cancer [5].

Interleukin-6 (IL-6) is a pleiotropic cytokine involved in tumor initiation, promotion, and progression [6]. IL-6 has been reported to be indispensable for oncogene-induced cell transformation and tumorigenesis, indicating the importance of IL-6 in tumor initiation [7], [8]. IL-6 deficiency has attenuated tumor development in a colitis-associated carcinogenesis model, demonstrating its role in inflammation-associated tumor promotion [9], [10]. IL-6 has also been reported to influence invasiveness and metastasis in various experimental models, suggesting its involvement in cancer progression [11], [12], [13], [14], [15].

Previous studies have suggested that IL-6 functions as a tumor-promoting factor in gastric cancer. Several studies examining IL-6 expression in human gastric cancer tissues showed that IL-6 expression was positively correlated with vascular endothelial growth factor (VEGF) expression, as well as tumor vascularity and histological grade [16], [17]. Other studies analyzing serum IL-6 levels in patients with gastric cancer revealed that a higher serum IL-6 level was an independent predictor of poor prognosis [18], [19]. However, the appropriate implementation of IL-6–targeted therapies requires further investigation of the mechanism underlying this association.

The roles of cancer-associated fibroblasts (CAFs) have been vigorously investigated in recent years. CAFs have been reported to promote tumor growth and invasion by inducing angiogenesis and changes in the extracellular matrix [20]. Most recently, IL-6 was revealed to be an important mediator in the interaction between tumor cells and CAFs in various experimental models, including a skin carcinogenesis mouse model [21], a co-cultivation system of human prostate epithelial cells and fibroblasts [22], and inflammation-induced gastric cancer mouse models [23].

To elucidate the role of IL-6 in gastric cancer, we examined IL-6 expression in human gastric cancer tissues. We also compared IL-6 knockout (IL-6^−/−^) mice with wild-type (WT) mice in a mouse model of chemically induced gastric tumorigenesis. Because these experiments showed that stromal fibroblasts expressed IL-6 in gastric cancer, we used primary human gastric fibroblasts *in vitro* to examine the role of IL-6 in epithelial–stromal interaction. We demonstrated that fibroblasts produced IL-6 in response to gastric cancer cells through IL-1 signaling, and that IL-6 promoted tumor growth through STAT3 activation.

## Materials and Methods

### Clinical Specimens

Gastric cancer specimens were obtained from the archives of Tokyo University Hospital (Tokyo, Japan) and Motojima General Hospital (Gumma, Japan) with the approval of the Ethics Committee of Graduate School of Medicine, the University of Tokyo or the Ethical Committee of Motojima General Hospital, and the acquisition of written informed consent from each patient.

### Cells and Reagents

Human cancer cell lines (SH101, AGS, NUGC4, MKN45, MKN74, and TMK1) were cultured in Ham’s F-12 medium, Dulbecco’s minimal essential medium (DMEM), or Roswell Park Memorial Institute (RPMI) medium supplemented with 10% fetal bovine serum (FBS). MKN45 and MKN74 were obtained from the Riken Gene Bank (Tsukuba, Japan). AGS was obtained from American Type Culture Collection (Manassas, VA). NUGC4 was obtained from the Cell Resource Center for Biomedical Research (Tohoku.University, Sendai, Japan). SH101 and TMK1 were provided by Dr. Tahara (Hiroshima University, Hiroshima, Japan) [24], [25]. Human gastric fibroblasts were isolated from adult human stomach specimens and cultured as described previously [26]. To verify the purity of the fibroblasts, we performed immunostaining of αSMA in the fibroblasts, showing all of the cells were positively stained with αSMA. Cell numbers were determined using a cell counting kit according to the manufacturer’s protocol (Dojindo Laboratories, Kumamoto, Japan).

We used recombinant human IL-6 (PeproTech Inc., Rocky Hill, NJ, USA), recombinant human IL-1 receptor antagonist (RA; R&D Systems Inc., Minneapolis, MN, USA), the Janus-activated kinase (JAK) inhibitor pyridone 6 (P6; Calbiochem, Darmstadt, Germany), and anti–tumor necrosis factor (TNF)-α neutralizing antibody (Remicade®; Centocor/Mitsubishi Tanabe Pharma, Osaka, Japan). Neutralizing anti-IL-6 receptor antibody (MRA) was kindly provided by Chugai [15].

### Preparation and Stimulation of Fibroblasts

Cancer cells were seeded and cultured in growth media containing 10% FBS for 24 h. After the cells were cultured in serum-free media for another 24 h, the supernatants were collected for enzyme-linked immunosorbent assay (ELISA) or other analyses.

Fibroblasts were seeded in 24-well plates and cultured in growth media containing 10% serum for 24 h. After the cells were treated with standard media or cancer cell–conditioned media for another 24 h, the supernatants were collected for ELISA analyses.

### Small Interfering RNA, Transfection, and Reagents

RNA oligonucleotides were synthesized by Dharmacon, Inc. (Boulder, CO). All small interfering RNA (siRNA) transfections were performed with LipofectAMINE 2000 (Invitrogen Life Technologies, Carlsbad, CA).

### *In Vitro* Coculture of Gastric Cancer Cells and Fibroblasts

For indirect co-culture experiments, gastric cancer cells were seeded in 24-well plates and cultured in growth media containing 10% serum for 24 h. After the cells were co-cultured with fibroblasts using transwell culturing inserts (BD Biosciences) with or without anti-IL-6 receptor antibody for another 24 h, cell lysates of gastric cancer cells were collected for analyses.

### Animal Experiments

IL-6^−/−^ mice (Jackson Laboratories, Bar Harbor, ME, USA) and C57BL/6 WT mice (Clea Japan, Tokyo, Japan) were used. All experimental protocols were approved by the Ethics Committee for Animal Experimentation at the Graduate School of Medicine, the University of Tokyo or the Ethics Committee of the Institute for Adult Diseases, Asahi Life Foundation, Tokyo, Japan and conducted in accordance with the Guidelines for the Care and Use of Laboratory Animals of the Department of Medicine, the University of Tokyo, and the Institute for Adult Diseases, Asahi Life Foundation, Tokyo, Japan.

For the gastric tumorigenesis model, 6-week-old WT and IL-6^−/−^ mice were given drinking water containing 240 ppm of N-methyl-N-nitrosourea (MNU; Sigma, St. Louis, MO, USA) in alternate weeks for a total of 5 weeks, as described previously [27]. Forty weeks after starting MNU administration, the stomach was removed from each mouse and the number and largest diameters of tumors were measured as described previously [27].

### Immunoblotting and Immunohistochemistry

Immunoblotting and immunohistochemistry were performed as described previously [27]. The antibodies used were anti-STAT3, anti-phospho-STAT3, anti-phospho-IκBα, anti-phospho-JNK, anti-phospho-p38 (all from Cell Signaling, Boston, MA, USA), anti-IκBα (Santa Cruz Biotechnology, Santa Cruz, CA, USA), anti-β-actin (Sigma), anti-IL-6, (Abcam, Cambridge, UK), anti-CD11b (Abcam), anti-α-smooth muscle actin (αSMA) (DAKO Japan, Tokyo, Japan), and anti-CD68 (DAKO Japan). For immunofluorescence, the sections were incubated with primary antibodies, followed by secondary Alexa488 and Alexa555 immunoglobulin G (IgG) antibodies (Invitrogen, Carlsbad, CA, USA).

### ELISA

The concentrations of IL-6, IL-1α, and IL-1β in the culture supernatants and the tissue lysates were measured using ELISA kits according to the manufacturer’s instructions (R&D Systems Inc.).

### Quantitative Real-Time Polymerase Chain Reaction Analysis

Quantitative real-time polymerase chain reaction (qRT-PCR) assays were performed to measure IL-1α or IL-6 mRNA using an ABI PRISM 7000 Quantitative PCR system (Applied Biosystems, Foster City, CA, USA). Each sample was examined in triplicate, and the amount of product was normalized in relation to glyceraldehyde-3-phosphate dehydrogenase (GAPDH). Primer sequences are available on request.

### Statistical Analysis

Data are expressed as means ± standard deviations (SD). Differences were analyzed by Student’s *t*-test. *P*<0.05 was deemed to indicate statistical significance.

## Results

### Stromal Fibroblasts Express IL-6 in Gastric Cancer

**Figure 1:**
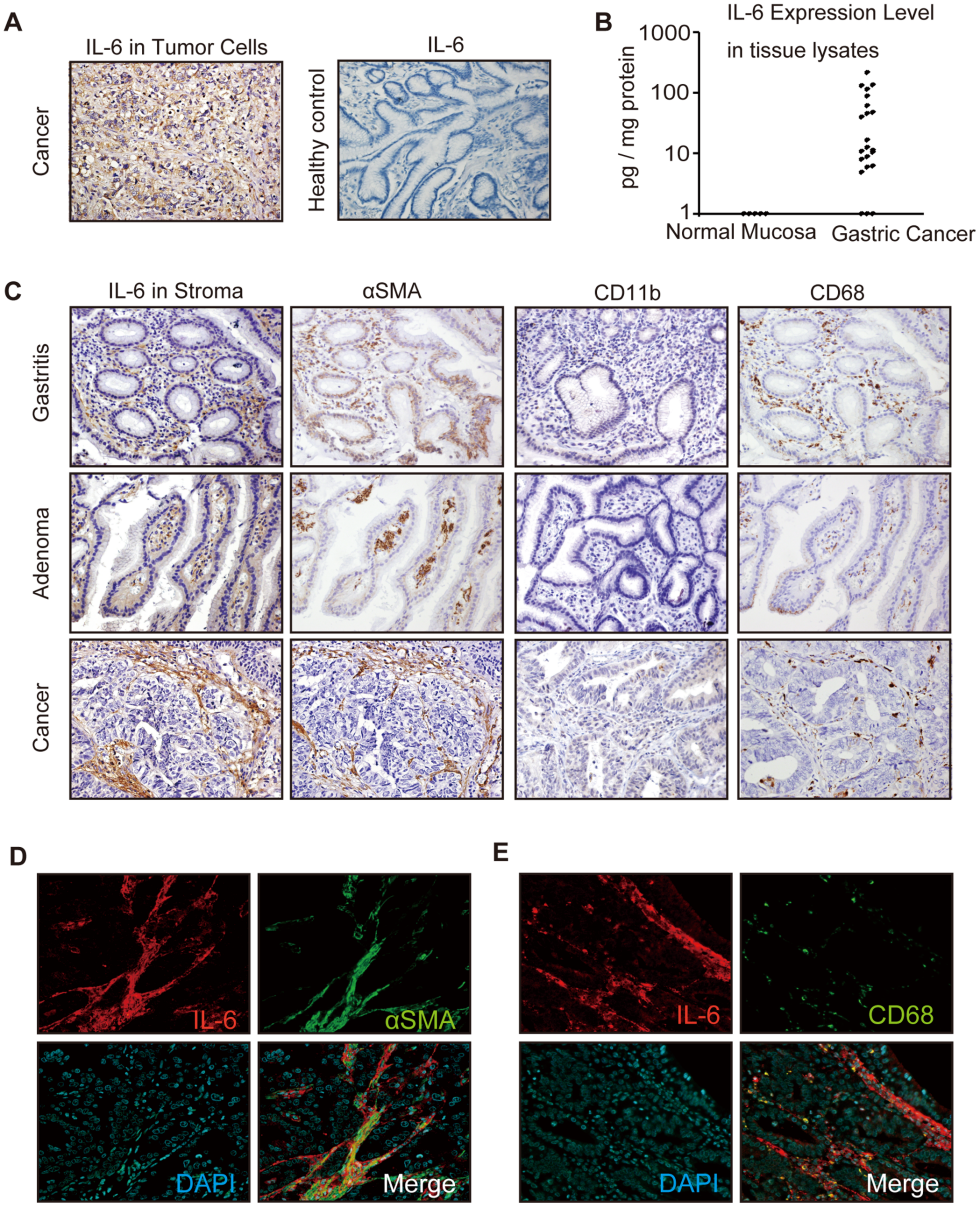
Interleukin-6 (IL-6) expression in gastric cancer tissues. (A) Representative pictures of immunohistochemical analyses of IL-6 in gastric cancer and *Helicobacter pylori*–negative healthy control (original magnification, ×200). (B) Enzyme-linked immunosorbent assay analysis of IL-6 expression in human gastric cancer tissues and normal gastric mucosa. (C) Representative pictures of immunohistochemical analyses of IL-6, α-smooth muscle actin (αSMA), CD11b, and CD68 in *Helicobacter pylori*–positive gastritis, gastric adenoma, and gastric cancer (original magnification, ×200). (D) Representative double staining of IL-6 (red) and αSMA (green) in human gastric cancer tissue (original magnification, ×200). (E) Representative double staining of IL-6 (red) and CD68 (green) in human gastric cancer tissue (original magnification, ×200).

To explore the involvement of IL-6 in gastric carcinogenesis, we performed an immunohistochemical analysis of IL-6 in 5 cases of *Helicobacter pylori*–negative healthy control, 5 cases of *Helicobacter pylori*–positive gastritis, 5 cases of gastric adenoma, and 31 cases of gastric cancer. None of gastric adenoma, gastritis, or healthy controls was positively stained for IL-6 in the epithelial cells. In 35.5% of gastric cancer cases, the tumor cells were positively stained for IL-6 expression (Fig. 1A). In contrast, the stroma was positively stained for IL-6 expression in none of healthy controls (Fig. 1A), 40% of gastritis cases, 20% of gastric adenoma, and 74.2% of gastric cancer cases (Fig. 1C). We also compared the IL-6 expression levels in gastric cancer tissues from 21 patients with those in *Helicobacter pylori*–negative normal gastric mucosa tissue samples from 6 healthy individuals by ELISA analysis (Fig. 1B). Of the 21 gastric cancer cases examined, 19 (86%) expressed IL-6 (4.9–220 pg/mg protein), whereas no normal gastric mucosa sample expressed a detectable level of IL-6.

Well-differentiated adenocarcinomas tended to express IL-6 in the stroma, whereas poorly differentiated adenocarcinomas and signet ring cell carcinomas were often positively stained in the tumor cells, although this tendency was not statistically significant (Table 1).

**Table 1:** Rate of interleukin-6 positive staining by degree of tumor differentiation.

	Well/Mod(n = 17)	Por/Sig(n = 14)	Total (n = 31)
IL-6 positive in fibroblasts	14 (82.4%)	9 (64.3%)	23 (74.2%)
IL-6 positive in tumor cells	5 (29.4%)	6 (42.9%)	11 (35.5%)

Well/Mod, well or moderately differentiated adenocarcinoma. Por/Sig, poorly differentiated adenocarcinoma or signet ring cell carcinoma.

Because of their spindle-shaped morphology, we hypothesized that the IL-6–positive cells in the stroma were fibroblasts. By performing immunostaining of αSMA (Fig. 1C) and double immunofluorescent staining of IL-6 and αSMA (Fig. 1D), we demonstrated that many IL-6–positive cells in the stroma were also αSMA-positive. This result suggests that stromal fibroblasts express IL-6.

To examine whether other immune cells such as myeloid–derived suppressor cells (MDSCs) and macrophages were also IL-6–positive cell population in the gastric tumor stroma, we performed an immunohistochemical analysis of CD11b and CD68 in the same samples (Fig. 1C). Although specific markers for human MDSCs are not well established, we used CD11b as a putative marker for MDSCs [28]. The results demonstrated that CD11b positive cells are scarce in human gastric tissues and that the morphology and distribution of CD68–positive macrophages differed from those of IL-6–positive cells. Immunofluorescent double staining of IL-6 and CD68 (Fig. 1E) demonstrated that only a small fraction of the IL-6–positive cells were also CD68-positive.

These results suggest that IL-6 is produced in the stroma of many gastric cancers and that fibroblasts are the major source of stromal IL-6.

### IL-6 Deficiency Attenuates MNU-induced Gastric Tumorigenesis in Mice

**Figure 2:**
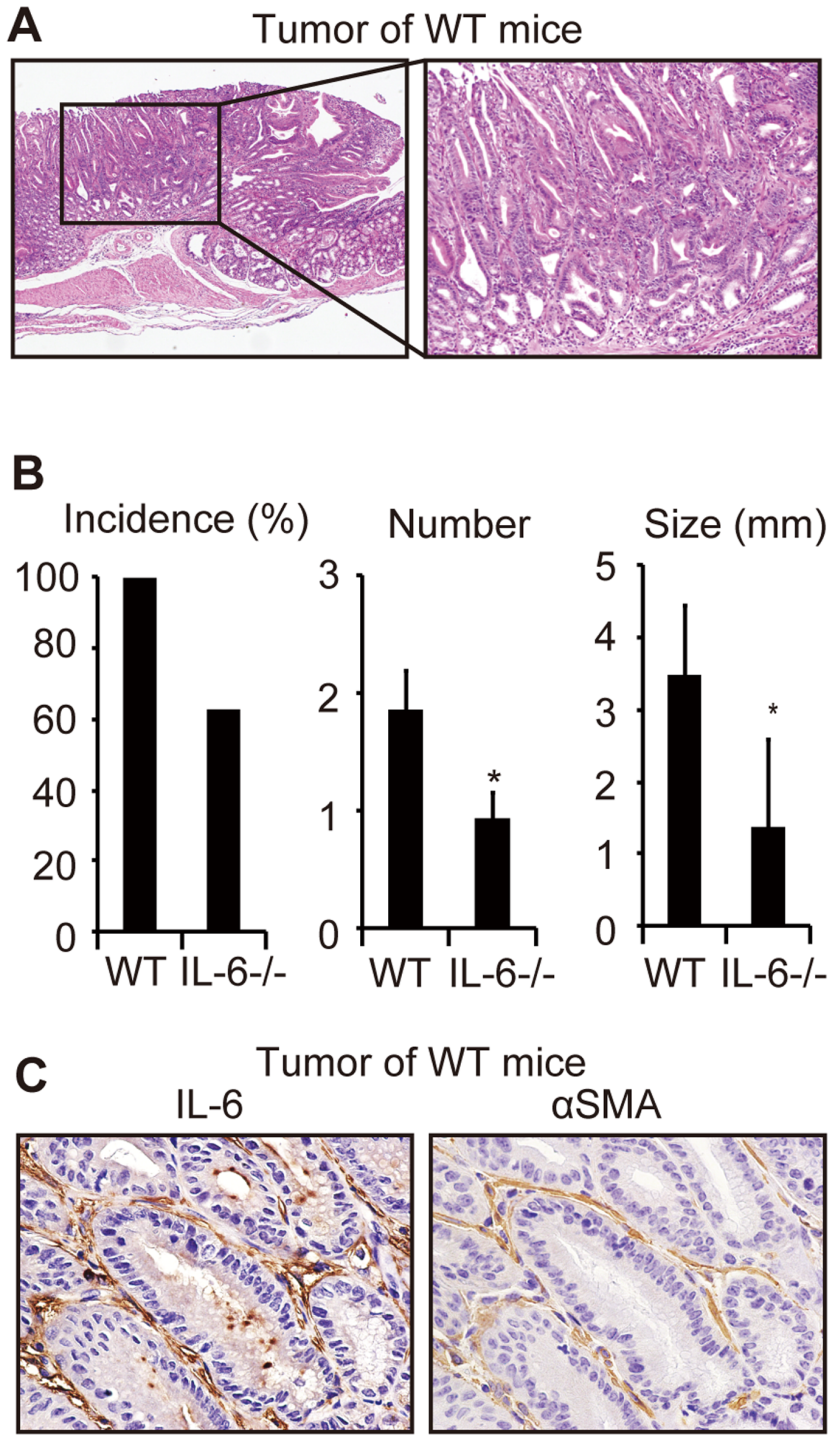
Interleukin-6 (IL-6) deficiency attenuated N-methyl-N-nitrosourea (MNU)-induced gastric tumorigenesis in mice. (A) Representative images of hematoxylin & eosin (H&E) staining of MNU-induced gastric tumors from wild-type (WT) mice [original magnification, ×40 (left), ×200 (right)]. (B) Tumor incidence rates, numbers of tumors, and maximal diameters of tumors. Values represent the means (± standard deviation) for WT (*n*  = 14) and IL-6^−/−^ (*n*  = 16) mice. *P<0.05 compared with WT mice. (C) Representative images of IL-6 (left) and α-smooth muscle actin (right) staining in a gastric tumor from a WT mouse (original magnification, ×200).

To examine the role of IL-6 in gastric tumorigenesis *in vivo*, we used a mouse model of gastric tumorigenesis induced by the chemical carcinogen MNU. A representative tumor in a WT mouse is shown in Fig. 2A.

All WT mice, but only 9/16 (56%) IL-6^−/−^ mice, developed gastric tumors (Fig. 2B). The number of detectable tumors and maximal tumor diameters were also significantly smaller in IL-6^−/−^ mice (Fig. 2B). These results suggest that IL-6 promotes gastric tumorigenesis.

Next, to test whether stromal fibroblasts expressed IL-6 in this model, we performed an immunohistochemical analysis of IL-6 and αSMA in the mouse gastric tumors (Fig. 2C). The localizations of IL-6 and αSMA in many of the same cells were similar to those seen in human gastric cancers, suggesting that stromal fibroblasts expressed IL-6 in the MNU-induced tumorigenesis mouse model.

### IL-6 Promotes Gastric Tumorigenesis and Tumor Growth through STAT3 Activation

**Figure 3:**
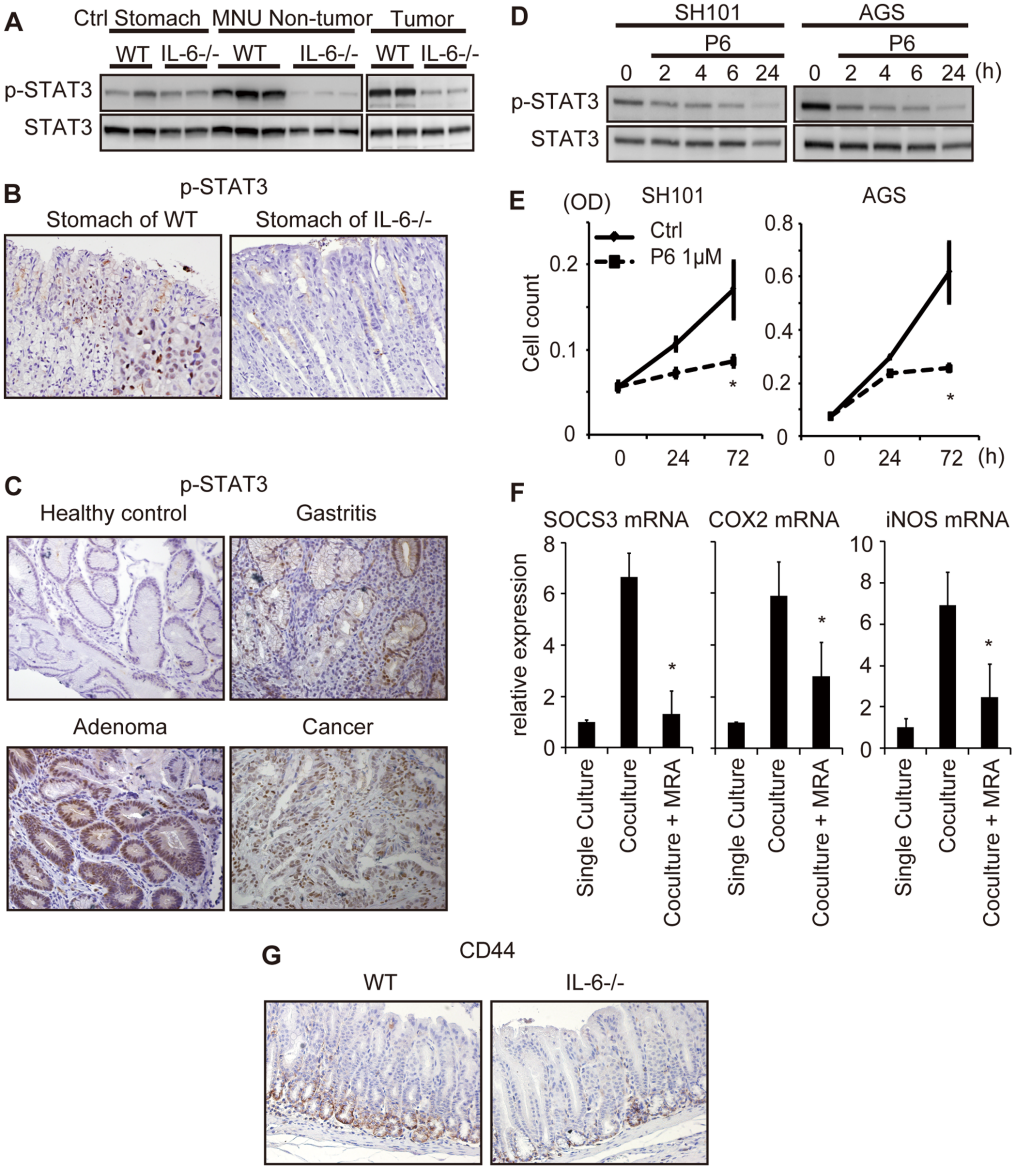
Interleukin-6 (IL-6) promoted gastric tumor development through STAT3 activation. (A) Immunoblot analysis of phosphorylated and total STAT3 in non-tumor tissues and tumors from wild-type (WT) and IL-6^−/−^ mice in the N-methyl-N-nitrosourea (MNU)-induced gastric tumorigenesis model. (B) Immunohistochemical analysis of phosphorylated STAT3 in WT (left) and IL-6^−/−^ (right) mice in the MNU-induced gastric tumorigenesis model. (C) Immunohistochemical analysis of phosphorylated STAT3 in *Helicobacter pylori*–negative healthy control, *Helicobacter pylori*–positive gastritis, gastric adenoma, and gastric cancer (original magnification, ×200). (D) Immunoblot analysis of phosphorylated and total STAT3 in SH101 and AGS cells treated with pyridone 6 (P6; 1 µM) for the indicated times. (E) SH101 and AGS cells were cultured with or without P6 (1 µM), and cell numbers were determined at the indicated times. Data are plotted as means (± standard deviations). *P<0.05 compared with cells without P6. (F) qRT-PCR for the indicated genes in gastric cancer cell line NUGC4 when co-cultured with fibroblasts with or without neutralizing anti-IL-6 receptor antibody (MRA). *P<0.05 compared with co-culture without MRA. (G) Immunohistochemical analysis of CD44 in stomach tissues of WT and IL-6−/− mice in chemically-induced gastric tumorigenesis model. (original magnification, ×200).

Next, we compared the phosphorylation activation of STAT3, an important downstream mediator of IL-6 signaling, in stomachs from MNU-treated WT and IL-6^−/−^ mice. Immunoblot analysis revealed that the phosphorylation of STAT3 was stronger in the stomachs of MNU-treated WT mice than in those of untreated control WT mice. However, this STAT3 activation was not observed in tumor or non-tumor tissues from MNU-induced IL-6^−/−^ mice (Fig. 3A). Similarly, immunoreactivity for phosphorylated STAT3 was stronger in stomach tissues from MNU-treated WT mice than in those from MNU-treated IL-6^−/−^ mice (Fig. 3B). These results suggest that IL-6 is important for the activation of STAT3 in gastric tumorigenesis.

We also performed an immunohistochemical analysis of phosphorylated STAT3 in 5 cases of *Helicobacter pylori*–negative healthy control, 5 cases of *Helicobacter pylori*–positive gastritis, 5 cases of gastric adenoma, and 5 cases of gastric cancer. While none of healthy controls was positively stained with phosphorylated STAT3, all cases of gastritis, gastric adenoma, gastric cancer, was positively stained for phosphorylated STAT3 in the epithelial cells (Fig. 3C). In human samples, not all of the phosphorylated STAT3 positive cases were IL-6 positive, indicating that pathways other than IL-6–STAT3 pathway are involved in some cases.

Next, we analyzed the significance of STAT3 activation in gastric cancer cells. Gastric cancer cell lines SH101 and AGS were cultured with or without the JAK inhibitor P6 [29]. P6 treatment inhibited constitutive STAT3 phosphorylation in SH101 and AGS cells (Fig. 3D). Cell proliferation was significantly inhibited by P6 (Fig. 3E), indicating that STAT3 activation in gastric cancer cells is involved in cell proliferation.

To analyze further how fibroblasts promote tumor progression through IL-6 signaling, we examined the expression of STAT3–related genes by qRT-PCR in gastric cancer cell line NUGC4 when co-cultured with fibroblasts with or without neutralizing anti-IL-6 receptor antibody (MRA). Among the 12 genes examined, SOCS3, COX2, and iNOS were induced in gastric cancer cells when co-cultured with fibroblasts (Fig. 3F). MRA attenuated this response, indicating that IL-6–STAT3 pathway is involved in the interaction between gastric cancer cells and fibroblasts. Since both COX2 and iNOS have been reported to be involved in gastric cancer promotion [30], induction of these genes can be one mechanism of tumor promotion by fibroblasts. The genes that were not significantly changed in response to fibroblasts include MCL1, BCL2, survivin, MMP7, SOCS1, MYC, IL-6, IL-8, and IL-11.

### IL-6 Affects Stem Cell Compartment in Gastric Tumorigenesis Model

Next, to examine if IL-6 affects stem cell compartment in gastric tumorigenesis, we performed immunohistochemical analysis of CD44, which has been proposed to be a stem cell marker in gastric tumorigenesis [31], in non-tumor part of stomach obtained from WT and IL-6−/− mice 40 weeks after starting MNU administration. We found that the expression level of CD44 in WT mice was significantly higher than in IL-6−/− mice (Fig. 3G). This result suggest that the effect of IL-6 on stem cell compartment could be one of the mechanism by which IL-6 promotes gastric tumorigenesis.

### Gastric Cancer Cells Stimulate Gastric Fibroblasts to Produce IL-6

**Figure 4:**
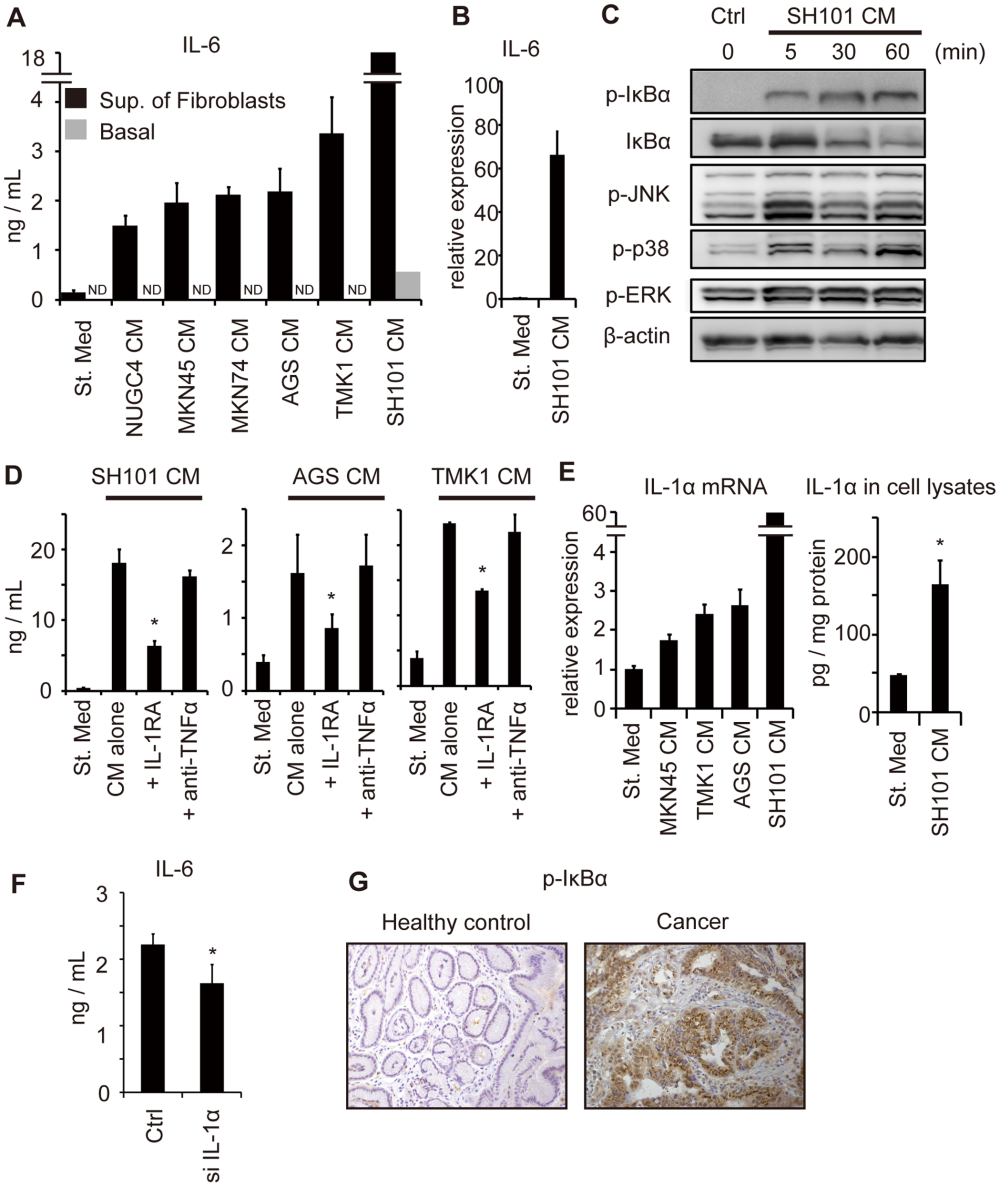
Gastric fibroblasts produced interleukin-6 (IL-6) in response to gastric cancer cell–conditioned media. (A) IL-6 concentrations in the supernatants of fibroblasts (black bars) and in the indicated conditioned media (gray bars). Fibroblasts were cultured with the indicated cancer cell–conditioned media or standard media. St. Med, standard media. CM, conditioned media. Sup. of Fibroblasts, supernatants of fibroblasts cultured with indicated media. ND, not detected. (B) qRT-PCR analysis of IL-6 expression in fibroblasts treated with conditioned media from SH101 cells. *P<0.05 compared with standard media. (C) Immunoblot analysis of the indicated proteins from the fibroblasts treated with SH101 cell–conditioned media for the indicated times. (D) IL-6 concentrations in the supernatants of fibroblasts treated with the indicated cancer cell–conditioned media with or without IL-1 receptor antagonist (100 ng/mL) or anti–tumor necrosis factor (TNF)α neutralizing antibody (100 µg/mL). *P<0.05 compared with conditioned media alone. (E) qRT-PCR (left) and enzyme-linked immunosorbent assay (right) for IL-1α in fibroblasts treated with the indicated cancer cell–conditioned media. *P<0.05 compared with standard media. (F) IL-6 concentrations in the supernatants of control or IL-1α siRNA transfected fibroblasts treated with the cancer cell–conditioned media. *P<0.05. (G) Immunohistochemical analysis of phosph-IκBα in stromal–IL-6 positive gastric cancer and *Helicobacter pylori*–negative healthy control (original magnification, ×200).

To examine the mechanisms of IL-6 production by fibroblasts, we used ELISA analysis to measure IL-6 produced in normal primary human gastric fibroblasts cultured *in vitro* in the presence or absence of conditioned media from cultured gastric cancer cells. The concentration of IL-6 in the supernatant of gastric fibroblasts under normal culture conditions was about 0.2 ng/mL. In contrast, the fibroblasts secreted 5–600 times more IL-6 when treated with cancer cell–conditioned media (Fig. 4A). The cancer cell–conditioned media alone did not explain the observed increase in IL-6 levels, because the IL-6 concentration was about 0.5 ng/mL in cancer cell–conditioned media from SH101 cells and undetectable in the other cell lines (Fig. 4A). These results demonstrate that the cancer cell–conditioned media stimulated IL-6 production in fibroblasts. Using qRT-PCR, we also examined the amount of IL-6 mRNA produced in fibroblasts that had been stimulated with SH101-conditioned media. The results showed that IL-6 mRNA was induced in fibroblasts when stimulated with cancer cell–conditioned media (Fig. 4B). These results demonstrate that the cancer cell–conditioned media stimulated IL-6 production in fibroblasts.

To elucidate the mechanism by which cancer cells activate fibroblasts, we examined the signal transduction pathways associated with IL-6 expression by immunoblot analysis (Fig. 4C). Phosphorylation and degradation of IκBα, as well as phosphorylation of JNK, p38, and extracellular receptor kinase (ERK), were observed after incubation for 5–60 min in cancer cell–conditioned media.

TNF receptors and IL-1 receptor are known to be important in the simultaneous activation of IκBα, JNK, and p38. To explore the involvement of TNF and IL-1, we added anti-TNFα neutralizing antibody and IL-1RA to the cancer cell–conditioned media prior to fibroblast stimulation. IL-6 secretion from fibroblasts stimulated with conditioned media from three different gastric cancer cell lines was significantly suppressed by IL-1RA but not by anti-TNFα (Fig. 4D), suggesting the involvement of IL-1 in the mechanisms of IL-6 secretion from fibroblasts stimulated by cancer cells.

To determine whether gastric cancer cells secreted IL-1α or IL-1β to activate fibroblasts, we measured the concentrations of IL-1α and IL-1β in cancer cell–conditioned media. However, neither IL-1α nor IL-1β was detected in the supernatants of NUGC4, MKN45, MKN74, AGS, or TMK1 cells. Only a small amount of IL-1α (10 pg/mL) was detected in the SH101-conditioned media. These data indicate that the mechanism of fibroblast suppression by IL-1RA is independent of IL-1 secretion by cancer cells.

Next, we considered the possibility that IL-1α or IL-1β stimulated fibroblasts in an autocrine fashion. However, neither IL-1α nor IL-1β was detected in the supernatants of gastric fibroblasts under normal conditions or when incubated with cancer cell–conditioned media.

IL-1α is known to be active mainly in its cell-associated forms, whereas IL-1β is solely active as a secreted product [32]. To examine whether cell-associated IL-1α of fibroblasts was involved in IL-6 upregulation, we measured the expression levels of IL-1α in fibroblast lysates by qRT-PCR and ELISA. The results showed that mRNA expression and IL-1α production were induced in fibroblasts when treated with cancer cell–conditioned media (Fig. 4E). Interestingly, the expression of IL-1α mRNA in fibroblasts by various cancer cell–conditioned media was comparable with IL-6 production by the same conditioned media (Fig. 4A). We also showed that siRNA inhibition of IL-1α in the fibroblasts significantly attenuated IL-6 production in response to the treatment with SH101–conditioned media (Fig. 4F). These data imply that cell-associated IL-1α is involved in the mechanisms of IL-6 production by fibroblasts.

Since IL-6 is a known target gene of NFκB signaling, we examined if tumor enhancement mediated by IL-6 positive fibroblasts is NFκB dependent. Immunostaining of phopho-IκBα in stromal IL-6–positive gastric cancer tissues showed that NFκB is activated both in tumor cells and in stromal cells (Fig. 4G).

## Discussion

**Figure 5:**
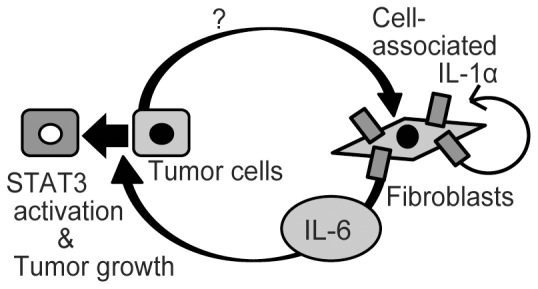
The proposed model of interaction between fibroblasts and tumor cells in gastric tumorigenesis.

In this study, we demonstrated that IL-6 plays an important role in the epithelial–stromal interaction in gastric tumorigenesis. We showed that stromal fibroblasts were the major source of IL-6, both in human gastric cancers and in chemically induced mouse gastric tumors. We also revealed that cancer cells affect IL-6 production from stromal fibroblasts through IL-1 signaling and that IL-6 is important for tumor growth through STAT3 activation (Fig. 5).

To our knowledge, this is the first report that focuses on the importance of IL-6 in gastric stromal fibroblasts. Previous studies of IL-6 in gastric cancer have examined only IL-6 expression in tumor cells or IL-6 levels in the serum of patients with gastric cancer [17], [18], [19]. Using mouse models of inflammation-induced gastric cancer, Quante et al. recently reported that CAFs could be derived from bone marrow mesenchymal stem cells [23]. The authors also revealed that stromal cell-derived factor (SDF)-1 and transforming growth factor (TGF)-β activated fibroblasts to express various cytokines, including IL-6. Our data suggest the involvement of IL-1 in fibroblast activation, which may be related to the marked accumulation of αSMA-positive fibroblasts demonstrated in the stomachs of IL-1β transgenic mice in their study [23].

The finding that IL-1 is involved in the activation of fibroblasts is compatible with other previous reports, such as a mouse skin carcinogenesis model in which immune cells were found to secrete IL-1β to activate fibroblasts [21], and *in vitro* analyses of senescent cells that have demonstrated that cell-surface bound IL-1α is an upstream regulator of IL-6 expression [33]. Previously, we reported that NFκB activity in gastric epithelial cells promoted proliferation and anti-apoptotic signaling via IL-1 [34]. In the current study, we revealed that IL-1 as well as NFκB activity in stromal fibroblasts promoted gastric tumorigenesis as a mediator of epithelial–stromal interaction. Because IL-1 is involved in a variety of mechanisms to enhance gastric tumorigenesis, it is an attractive therapeutic target for gastric cancer.

Although most experimental studies of CAFs have highlighted their functions in cancer promotion and progression, such as angiogenesis and invasion, we speculated that stromal fibroblasts might promote gastric tumorigenesis from an earlier stage. In our mouse model, MNU-induced gastric tumors were not invasive cancers, but were dysplasias or intramucosal cancers [34]. Immunohistochemical analysis of IL-6 in human samples indicated that IL-6 was expressed in the stroma of some cases of gastritis and adenoma. Thus, it is probable that IL-6 derived from stromal fibroblasts promotes tumorigenesis from an early stage of the gastric tumor. This notion is consistent with the model of Erez et al., in which CAFs were activated in the initial hyperplastic stage of multistep skin tumorigenesis [21].

The degree of stromal fibroblast contribution to tumorigenesis may differ according to the degree of tumor differentiation. As shown in Table 1, well-differentiated adenocarcinomas tend to express IL-6 in stromal fibroblasts. Lee et al. also reported that the histological differentiation of adenocarcinomas was related to IL-6 expression in epithelial cells, although they did not analyze stromal IL-6 [16].

IL-6– and IL-1–targeted therapies have been used clinically to treat various diseases, such as rheumatoid arthritis [35], [36] and Castleman’s disease [37]. These therapies may have additional clinical applications in the treatment of gastric cancer. Based on our results, future IL-6– and IL-1–targeted therapies might be expected to exert their action not only on gastric tumor cells, but also on stromal fibroblasts.

To date, suggested mechanisms of tumor promotion by IL-6-STAT3 pathway include support of cell survival, enhancement of migration and invasion, and elevation in angiogenic phenotype [38], [39], [40]. In *in vitro* co-culture experiment, we found that iNOS and COX-2, which were reported to be involved in gastric cancer promotion, were induced in gastric cancer cells through interaction with fibroblasts in IL-6-STAT3-pathway dependent manner. This result suggests those genes as IL-6-STAT3-pathway related effecters in tumor promotion.

In a string of studies using gp130 mutant mouse model of gastric cancer, the importance of IL-11 was highlighted and IL-6 is reported to be relatively unimportant [41], [42], [43]. The inconsistency between these reports and our results may be explained by the difference of the experimental model. In gp130 mutant mice, constitutively active STAT3 and IL-11 constitute a positive feedback loop, which is the main mechanism of gastric tumorigenesis. In this model, IL-6 may compete with IL-11 for the same receptor gp130 and interfere in the positive feedback loop. In chemically-induced gastric tumorigenesis model, pathways other than IL-11-STAT3 pathway, including JNK and NFκB pathways, also contribute to gastric tumorigenesis [34], [44]. IL-6-STAT3 pathway can contribute to tumor promotion under these conditions.

Effects on cancer stem cell compartment may be one of the mechanisms of tumor promotion through IL-6. Recent study showed that IL-6 had the ability to induce CD44 positive cancer stem cells in breast cancer oncogenesis model [45]. Other group reported that CD44 was a good marker of gastric cancer stem cell [31]. In the present study, we showed that IL-6 deficiency attenuated the expression of CD44 in *in vivo* gastric tumorigenesis model, suggesting the relationship among IL-6, CD44, and cancer stem cells.

We found that gastric cancer cell–conditioned media stimulated fibroblasts to produce IL-6, but we did not identify the specific molecules in the media responsible for this stimulation. In a mouse model of spontaneous gastric tumorigenesis induced by the simultaneous activation of prostaglandin E2 and Wnt signaling, gastric fibroblasts required stimulation by tumor cells to express angiogenic factors, although the specific molecules secreted by tumor cells were not identified [46]. TGF-β, which is known to mediate the transition of normal fibroblasts into CAFs [20], might be a major candidate because TGF-β from scirrhous gastric cancer cells activates fibroblasts and promotes collagen synthesis of fibroblasts [47]. However, in our study, TGF-β treatment did not affect IL-6 production by gastric fibroblasts (data not shown). Thus, further investigation of this issue is required to identify a new therapeutic strategy.

In summary, gastric stromal fibroblasts produced IL-6 in response to stimulation from tumor cells through IL-1 signaling in gastric tumorigenesis, and that secreted IL-6 promotes tumor growth through STAT3 activation.

## References

[1] Parkin DM, Bray F, Ferlay J, Pisani P (2005) Global cancer statistics, 2002. CA Cancer J Clin 55: 74–108. 15761078 10.3322/canjclin.55.2.74

[2] Inoue M, Tsugane S (2005) Epidemiology of gastric cancer in Japan. Postgrad Med J 81: 419–424. 15998815 10.1136/pgmj.2004.029330 PMC1743301

[3] Koizumi W, Narahara H, Hara T, Takagane A, Akiya T, et al (2008) S-1 plus cisplatin versus S-1 alone for first-line treatment of advanced gastric cancer (SPIRITS trial): a phase III trial. Lancet Oncol 9: 215–221. 18282805 10.1016/S1470-2045(08)70035-4

[4] Lin W, Kao HW, Robinson D, Kung HJ, Wu CW, et al (2000) Tyrosine kinases and gastric cancer. Oncogene 19: 5680–5689. 11114748 10.1038/sj.onc.1203924

[5] Bang YJ, Van Cutsem E, Feyereislova A, Chung HC, Shen L, et al (2010) Trastuzumab in combination with chemotherapy versus chemotherapy alone for treatment of HER2-positive advanced gastric or gastro-oesophageal junction cancer (ToGA): a phase 3, open-label, randomised controlled trial. Lancet 376: 687–697. 20728210 10.1016/S0140-6736(10)61121-X

[6] Hong DS, Angelo LS, Kurzrock R (2007) Interleukin-6 and its receptor in cancer: implications for translational therapeutics. Cancer 110: 1911–1928. 17849470 10.1002/cncr.22999

[7] Ancrile B, Lim KH, Counter CM (2007) Oncogenic Ras-induced secretion of IL6 is required for tumorigenesis. Genes Dev 21: 1714–1719. 17639077 10.1101/gad.1549407 PMC1920165

[8] Iliopoulos D, Hirsch HA, Struhl K (2009) An epigenetic switch involving NF-kappaB, Lin28, Let-7 MicroRNA, and IL6 links inflammation to cell transformation. Cell 139: 693–706. 19878981 10.1016/j.cell.2009.10.014 PMC2783826

[9] Grivennikov S, Karin E, Terzic J, Mucida D, Yu GY, et al (2009) IL-6 and Stat3 are required for survival of intestinal epithelial cells and development of colitis-associated cancer. Cancer Cell 15: 103–113. 19185845 10.1016/j.ccr.2009.01.001 PMC2667107

[10] Bollrath J, Phesse TJ, von Burstin VA, Putoczki T, Bennecke M, et al (2009) gp130-mediated Stat3 activation in enterocytes regulates cell survival and cell-cycle progression during colitis-associated tumorigenesis. Cancer Cell 15: 91–102. 19185844 10.1016/j.ccr.2009.01.002

[11] Tang CH, Chen CF, Chen WM, Fong YC (2011) IL-6 increases MMP-13 expression and motility in human chondrosarcoma cells. J Biol Chem 286: 11056–11066. 21278254 10.1074/jbc.M110.204081 PMC3064160

[12] Lin MT, Lin BR, Chang CC, Chu CY, Su HJ, et al (2007) IL-6 induces AGS gastric cancer cell invasion via activation of the c-Src/RhoA/ROCK signaling pathway. Int J Cancer 120: 2600–2608. 17304514 10.1002/ijc.22599

[13] Walter M, Liang S, Ghosh S, Hornsby PJ, Li R (2009) Interleukin 6 secreted from adipose stromal cells promotes migration and invasion of breast cancer cells. Oncogene 28: 2745–2755. 19483720 10.1038/onc.2009.130 PMC2806057

[14] Yadav A, Kumar B, Datta J, Teknos TN, Kumar P (2011) IL-6 promotes head and neck tumor metastasis by inducing epithelial-mesenchymal transition via the JAK-STAT3-SNAIL signaling pathway. Mol Cancer Res 9(12): 1658–1667. 21976712 10.1158/1541-7786.MCR-11-0271 PMC3243808

[15] Maeda S, Hikiba Y, Sakamoto K, Nakagawa H, Hirata Y, et al (2009) Ikappa B kinasebeta/nuclear factor-kappaB activation controls the development of liver metastasis by way of interleukin-6 expression. Hepatology 50: 1851–1860. 19821485 10.1002/hep.23199

[16] Lee SA, Choi SR, Jang JS, Lee JH, Roh MH, et al (2010) Expression of VEGF, EGFR, and IL-6 in Gastric Adenomas and Adenocarcinomas by Endoscopic Submucosal Dissection. Dig Dis Sci 55(7): 1955–1963. 19757047 10.1007/s10620-009-0967-1

[17] Huang SP, Wu MS, Shun CT, Wang HP, Lin MT, et al (2004) Interleukin-6 increases vascular endothelial growth factor and angiogenesis in gastric carcinoma. J Biomed Sci 11: 517–527. 15153787 10.1007/BF02256101

[18] Liao WC, Lin JT, Wu CY, Huang SP, Lin MT, et al (2008) Serum interleukin-6 level but not genotype predicts survival after resection in stages II and III gastric carcinoma. Clin Cancer Res 14: 428–434. 18198221 10.1158/1078-0432.CCR-07-1032

[19] Ashizawa T, Okada R, Suzuki Y, Takagi M, Yamazaki T, et al (2005) Clinical significance of interleukin-6 (IL-6) in the spread of gastric cancer: role of IL-6 as a prognostic factor. Gastric Cancer 8: 124–131. 15864720 10.1007/s10120-005-0315-x

[20] Kalluri R, Zeisberg M (2006) Fibroblasts in cancer. Nat Rev Cancer 6: 392–401. 16572188 10.1038/nrc1877

[21] Erez N, Truitt M, Olson P, Arron ST, Hanahan D (2010) Cancer-Associated Fibroblasts Are Activated in Incipient Neoplasia to Orchestrate Tumor-Promoting Inflammation in an NF-kappaB-Dependent Manner. Cancer Cell 17: 135–147. 20138012 10.1016/j.ccr.2009.12.041

[22] Paland N, Kamer I, Kogan-Sakin I, Madar S, Goldfinger N, et al (2009) Differential influence of normal and cancer-associated fibroblasts on the growth of human epithelial cells in an in vitro cocultivation model of prostate cancer. Mol Cancer Res 7: 1212–1223. 19671672 10.1158/1541-7786.MCR-09-0073

[23] Quante M, Tu SP, Tomita H, Gonda T, Wang SS, et al (2011) Bone marrow-derived myofibroblasts contribute to the mesenchymal stem cell niche and promote tumor growth. Cancer Cell 19: 257–272. 21316604 10.1016/j.ccr.2011.01.020 PMC3060401

[24] Nishiyama M, Saeki S, Aogi K, Hirabayashi N, Toge T (1993) Relevance of DT-diaphorase activity to mitomycin C (MMC) efficacy on human cancer cells: differences in in vitro and in vivo systems. Int J Cancer 53: 1013–1016. 8473041 10.1002/ijc.2910530626

[25] Ochiai A, Yasui W, Tahara E (1985) Growth-promoting effect of gastrin on human gastric carcinoma cell line TMK-1. Jpn J Cancer Res 76: 1064–1071. 3003017

[26] Takahashi M, Kawabe T, Ogura K, Maeda S, Mikami Y, et al (1997) Expression of vascular endothelial growth factor at the human gastric ulcer margin and in cultured gastric fibroblasts: a new angiogenic factor for gastric ulcer healing. Biochem Biophys Res Commun 234: 493–498. 9177300 10.1006/bbrc.1997.5974

[27] Hayakawa Y, Hirata Y, Nakagawa H, Sakamoto K, Hikiba Y, et al (2011) Apoptosis signal-regulating kinase 1 and cyclin D1 compose a positive feedback loop contributing to tumor growth in gastric cancer. Proc Natl Acad Sci U S A 108: 780–785. 21187402 10.1073/pnas.1011418108 PMC3021038

[28] Montero AJ, Diaz-Montero CM, Kyriakopoulos CE, Bronte V, Mandruzzato S (2012) Myeloid-derived suppressor cells in cancer patients: a clinical perspective. J Immunother 35: 107–115. 22306898 10.1097/CJI.0b013e318242169f

[29] Pedranzini L, Dechow T, Berishaj M, Comenzo R, Zhou P, et al (2006) Pyridone 6, a pan-Janus-activated kinase inhibitor, induces growth inhibition of multiple myeloma cells. Cancer Res 66: 9714–9721. 17018630 10.1158/0008-5472.CAN-05-4280

[30] Chen CN, Hsieh FJ, Cheng YM, Chang KJ, Lee PH (2006) Expression of inducible nitric oxide synthase and cyclooxygenase-2 in angiogenesis and clinical outcome of human gastric cancer. J Surg Oncol 94: 226–233. 16900533 10.1002/jso.20372

[31] Takaishi S, Okumura T, Tu S, Wang SS, Shibata W, et al (2009) Identification of gastric cancer stem cells using the cell surface marker CD44. Stem Cells 27: 1006–1020. 19415765 10.1002/stem.30 PMC2746367

[32] Dinarello CA (1996) Biologic basis for interleukin-1 in disease. Blood 87: 2095–2147. 8630372

[33] Orjalo AV, Bhaumik D, Gengler BK, Scott GK, Campisi J (2009) Cell surface-bound IL-1alpha is an upstream regulator of the senescence-associated IL-6/IL-8 cytokine network. Proc Natl Acad Sci U S A 106: 17031–17036. 19805069 10.1073/pnas.0905299106 PMC2761322

[34] Sakamoto K, Hikiba Y, Nakagawa H, Hayakawa Y, Yanai A, et al.. (2010) Inhibitor of kappaB kinase beta regulates gastric carcinogenesis via interleukin-1alpha expression. Gastroenterology 139: 226–238 e226. 10.1053/j.gastro.2010.03.047 PMC3156098 20347815

[35] Sebba A (2008) Tocilizumab: the first interleukin-6-receptor inhibitor. Am J Health Syst Pharm 65: 1413–1418. 18653811 10.2146/ajhp070449

[36] Fleischmann RM, Tesser J, Schiff MH, Schechtman J, Burmester GR, et al (2006) Safety of extended treatment with anakinra in patients with rheumatoid arthritis. Ann Rheum Dis 65: 1006–1012. 16396977 10.1136/ard.2005.048371 PMC1798263

[37] Nishimoto N, Kanakura Y, Aozasa K, Johkoh T, Nakamura M, et al (2005) Humanized anti-interleukin-6 receptor antibody treatment of multicentric Castleman disease. Blood 106: 2627–2632. 15998837 10.1182/blood-2004-12-4602

[38] Kanda N, Seno H, Konda Y, Marusawa H, Kanai M, et al (2004) STAT3 is constitutively activated and supports cell survival in association with survivin expression in gastric cancer cells. Oncogene 23: 4921–4929. 15077160 10.1038/sj.onc.1207606

[39] Okamoto W, Okamoto I, Arao T, Yanagihara K, Nishio K, et al (2011) Differential roles of STAT3 depending on the mechanism of STAT3 activation in gastric cancer cells. Br J Cancer 105: 407–412. 21730976 10.1038/bjc.2011.246 PMC3172904

[40] Gong W, Wang L, Yao JC, Ajani JA, Wei D, et al (2005) Expression of activated signal transducer and activator of transcription 3 predicts expression of vascular endothelial growth factor in and angiogenic phenotype of human gastric cancer. Clin Cancer Res 11: 1386–1393. 15746037 10.1158/1078-0432.CCR-04-0487

[41] Ernst M, Najdovska M, Grail D, Lundgren-May T, Buchert M, et al (2008) STAT3 and STAT1 mediate IL-11-dependent and inflammation-associated gastric tumorigenesis in gp130 receptor mutant mice. J Clin Invest 118: 1727–1738. 18431520 10.1172/JCI34944 PMC2323192

[42] Howlett M, Judd LM, Jenkins B, La Gruta NL, Grail D, et al (2005) Differential regulation of gastric tumor growth by cytokines that signal exclusively through the coreceptor gp130. Gastroenterology 129: 1005–1018. 16143138 10.1053/j.gastro.2005.06.068

[43] Howlett M, Giraud AS, Lescesen H, Jackson CB, Kalantzis A, et al (2009) The interleukin-6 family cytokine interleukin-11 regulates homeostatic epithelial cell turnover and promotes gastric tumor development. Gastroenterology 136: 967–977. 19121317 10.1053/j.gastro.2008.12.003

[44] Shibata W, Maeda S, Hikiba Y, Yanai A, Sakamoto K, et al (2008) c-Jun NH2-terminal kinase 1 is a critical regulator for the development of gastric cancer in mice. Cancer Res 68: 5031–5039. 18593901 10.1158/0008-5472.CAN-07-6332

[45] Iliopoulos D, Hirsch HA, Wang G, Struhl K (2011) Inducible formation of breast cancer stem cells and their dynamic equilibrium with non-stem cancer cells via IL6 secretion. Proc Natl Acad Sci U S A 108: 1397–1402. 21220315 10.1073/pnas.1018898108 PMC3029760

[46] Guo X, Oshima H, Kitmura T, Taketo MM, Oshima M (2008) Stromal fibroblasts activated by tumor cells promote angiogenesis in mouse gastric cancer. J Biol Chem 283: 19864–19871. 18495668 10.1074/jbc.M800798200

[47] Mahara K, Kato J, Terui T, Takimoto R, Horimoto M, et al (1994) Transforming growth factor beta 1 secreted from scirrhous gastric cancer cells is associated with excess collagen deposition in the tissue. Br J Cancer 69: 777–783. 8142266 10.1038/bjc.1994.147 PMC1968800

